# Agenesis of dorsal pancreas associated with pancreatic neuroendocrine tumor: a case report and review of the literature

**DOI:** 10.1186/s13256-018-1733-9

**Published:** 2018-06-30

**Authors:** A. Erotokritou, C. D. Gerharz, A. Sagir

**Affiliations:** 1 Department of Gastroenterology, Academic Teaching Hospital Bethesda Duisburg, Heerstr, 219 47053 Duisburg, Germany; 2 Department of Pathology, Academic Teaching Hospital Bethesda Duisburg, Heerstr. 219, 47053 Duisburg, Germany

**Keywords:** Agenesis, Pancreas, Tumor, Neuroendocrine

## Abstract

**Background:**

Agenesis of the dorsal pancreas is very rare. Less than 70 cases have been reported to date. Some of these cases had an association with a tumor. The literature of agenesis of the dorsal pancreas and agenesis of the dorsal pancreas-associated pancreatic neoplasia is limited. Here we report the second case of a pancreatic neuroendocrine tumor in a setting of agenesis of the dorsal pancreas.

**Case presentation:**

A 71-year-old man, originally from North Africa, with a history of insulin-dependent diabetes mellitus, presented with a 2-month history of nonspecific abdominal symptoms. Contrast-enhanced computed tomography demonstrated an almost 3 cm round, quite well-defined and homogeneous tumor formation in the area between the neck and absent body and tail of his pancreas. The mass was confirmed by endoscopic ultrasound. Our patient underwent computed tomography-guided biopsy of the mass which provided proof of a neuroendocrine tumor. He underwent pancreas resection because of the presence of a neuroendocrine tumor. Seven months later his glycated hemoglobin increased from 6.9 to 8.7%.

**Conclusions:**

Diagnosis of agenesis of the dorsal pancreas is based on imaging techniques like computed tomography, magnetic resonance cholangiopancreatography, or endoscopic ultrasound. Endoscopic ultrasound-guided fine-needle aspiration can be helpful for the histological diagnosis of the tumor. The hypothesis of the association between pancreatic neoplasia and agenesis of the dorsal pancreas leads us to the suggestion that every patient with diagnosed agenesis of the dorsal pancreas should be observed with a focus on the early detection of potential malignancy.

## Background

Pancreatic development is a complex process with a fusion of the ventral and dorsal bud. Development anomalies of the pancreas have been described but agenesis of the dorsal pancreas (ADP) is very rare. Around 60 cases of ADP have been published up to 2017. More than ten reported cases of ADP were associated with a pancreatic tumor.

Here we report a rare case of ADP with a neuroendocrine tumor; only one case has been published before.

## Case presentation

A 71-year-old man, originally from North Africa, with a history of insulin-dependent diabetes mellitus, presented to our emergency department with a 2-month history of nonspecific abdominal symptoms: meteorism (bloating) and a subjective feeling of abdominal enlargement. Diarrhea, loss of appetite, weight loss, persistent fever, night sweats, headaches, anxiety, gastric ulcer disease, or skin rash were not reported. He did not report a neoplasm in the past and he had not undergone an abdominal examination before. He could not recall any episodes of pancreatitis or suffering from gall bladder stones. He had undergone a computed tomography (CT) scan a few days before which showed a tumor in the pancreatic head and he was referred to our clinic. Diabetes mellitus occurred 20 years ago and he was initially treated with orally administered anti-diabetic drugs for more than 12 years. Apart from diabetes and arterial hypertension he had no previous medical or surgical history. He has been treated with two different anti-hypertensives, two diuretics, acetylsalicylic acid, and insulin glargine. He reported an allergy to metformin. He did not smoke tobacco or drink alcohol.

### Physical examination

On general physical examination, he was conscious and oriented and in fair general condition. He appeared to be in a good nutritional state (height 165 cm, weight 73 kg, body mass index 26.8 kg/m^2^). His lungs were clear to auscultation and percussion bilaterally. His heart examination was also normal. He had a soft, non-tender abdomen without any palpable masses. Icterus and lymphadenopathy were absent. His vital signs were normal.

### Laboratory findings

A routine laboratory analysis showed the following abnormal values: hemoglobin 12.5 g/dl (13.5–17.5), hematocrit 25% (40–53), mean corpuscular volume (MCV) 78 fl (82–98), uric acid 10.2 mg/dl (< 7.0), creatinine 1.8 mg/dl (< 1.4), and random serum glucose 148 mg/dl. Transaminases and cholestasis parameter were normal. Glycated hemoglobin (HbA1c) was 6.9% (52 mmol/mol). The serum tumor markers carcinoembryonic antigen (CEA) and cancer antigen 19–9 (CA19–9) were in normal range. Serum pancreatic lipase was slightly elevated (88 U/l, reference range < 65 U/l). The pancreatic elastase in stool was normal. There was no clinical evidence for exocrine pancreatic insufficiency.

### Imaging modalities and histology

**Fig. 1:**
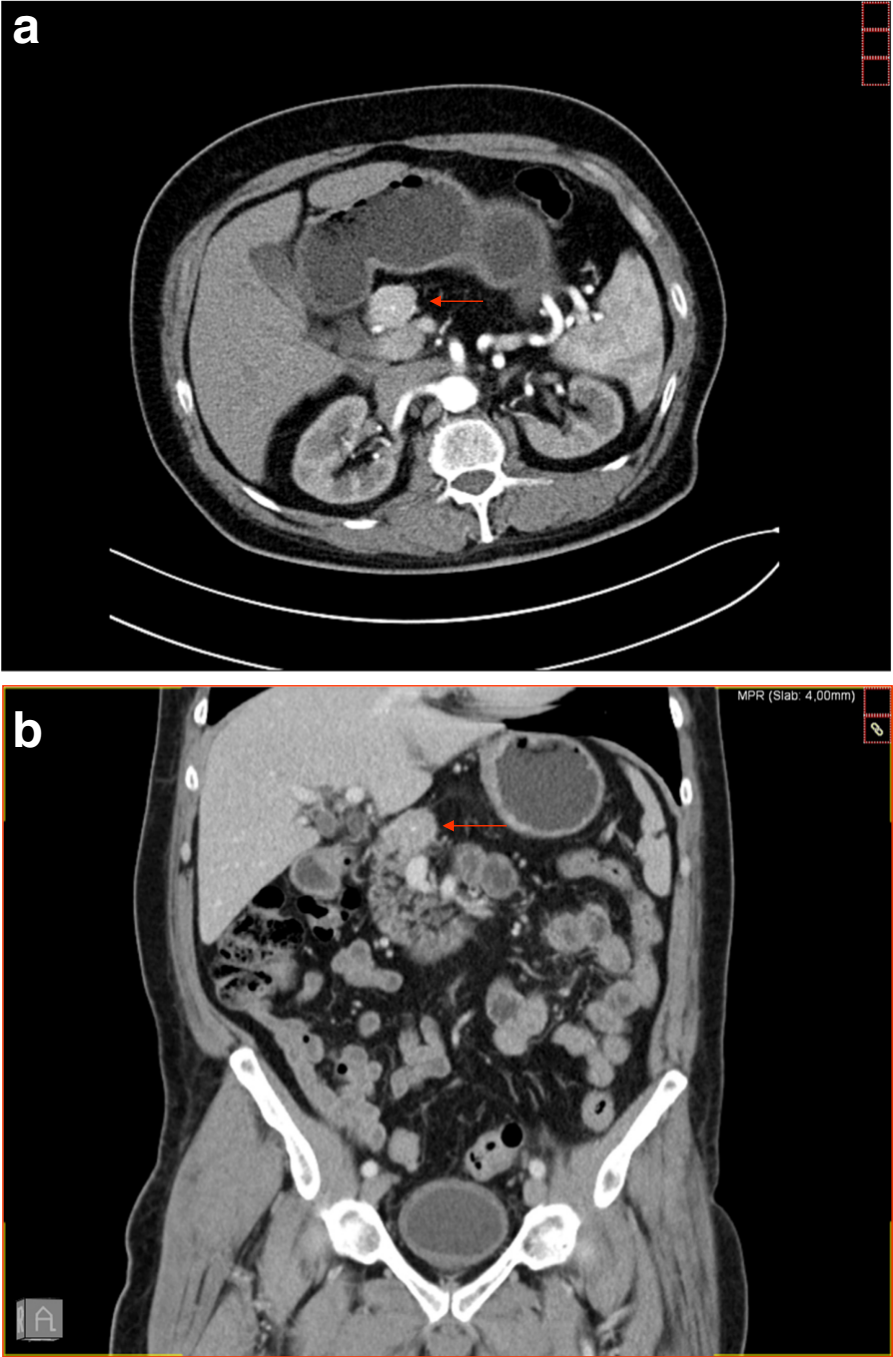
Contrast-enhanced computed tomography scan with axial image in arterial phase (**a**) and coronal reconstructed image in venous phase (**b**) showing a round hypervascular mass, measuring approximately 3 cm at the neck of the pancreas with absence of the body and tail of the pancreas. The *arrows* show the lesion in the pancreas

An ultrasound of his abdomen revealed a tumor of the uncinate process of the pancreas. The endoscopic ultrasound showed a mass in the area between the neck and the body of his pancreas, measuring approximately 24 mm in its largest dimension. The tumor compressed his splenic vein; however, there were no signs of infiltration. Contrast-enhanced CT demonstrated an almost 3 cm round, quite well-defined and homogeneous tumor formation in the area between the neck and absent body of his pancreas (Fig. 1). The tail of the pancreas was absent as well. The tumor showed a slightly arterial hypervascular enhancement and a central calcification in the shape of a dot. There was no central hypoperfusion or necrosis. The imaging findings were not typically suggestive of exocrine pancreatic carcinoma or pancreatic endocrine tumor. After discussing the case at our interdisciplinary tumor board, we decided to perform a biopsy.

The initial fine-needle aspiration was performed via endoscopic ultrasound. However, the pathological analysis did not detect tumor cells in the aspiration material.

**Fig. 2:**
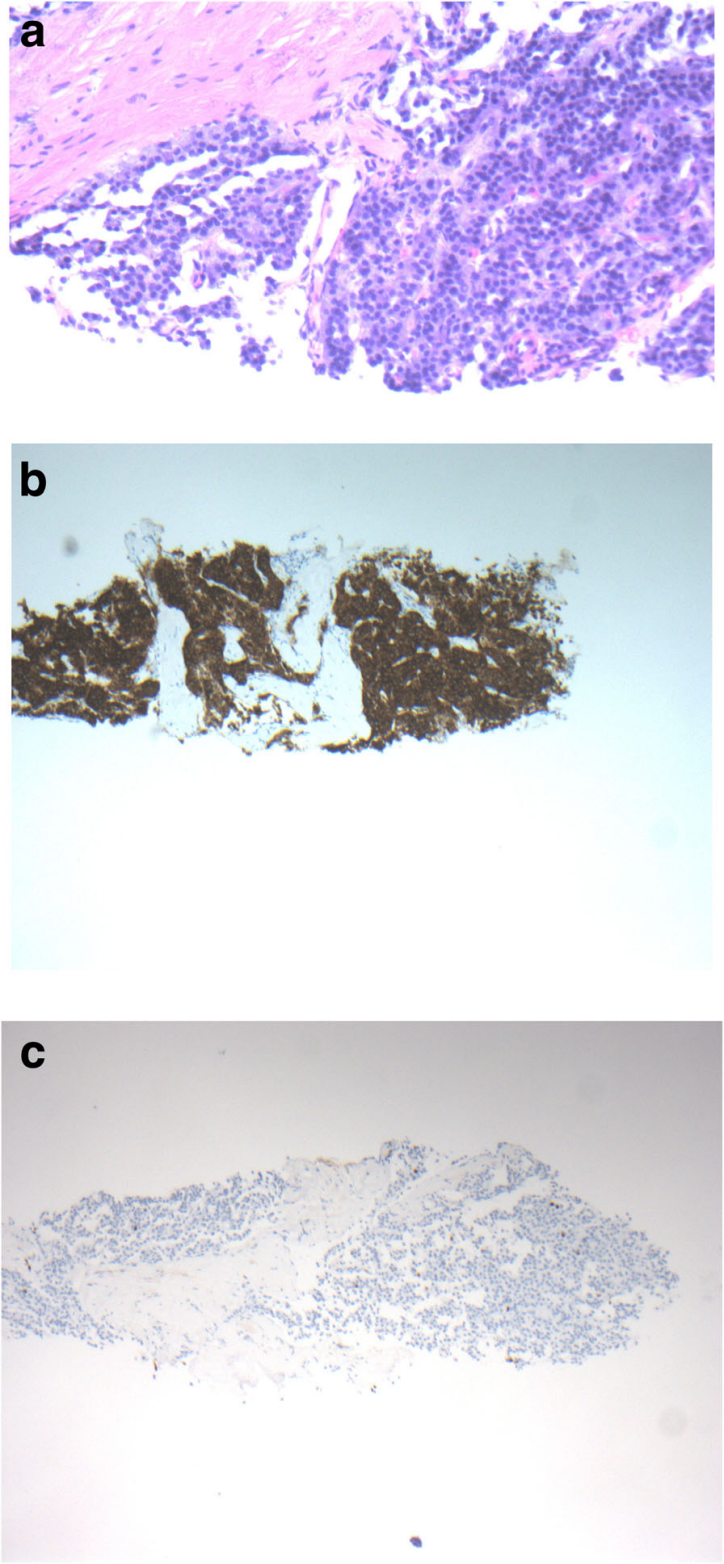
Well-differentiated neuroendocrine tumor with solid tumor cell aggregates; hematoxylin and eosin (**a**) exhibiting intensive cytoplasmic staining for synaptophysin (**b**), and nuclear staining for Ki-67 in less than 2% of the tumor cells (**c**)

In the next step our patient underwent CT-guided biopsy of the mass. A histopathological examination (Fig. 2a) revealed the typical aspect of a well-differentiated neuroendocrine tumor with solid aggregates of isomorphic tumor cells. Immunohistochemistry showed intensive cytoplasmic staining for synaptophysin (Fig. 2b) and nuclear staining for Ki-67 in less than 2% of the tumor cells (Fig. 2c). No gastrin expression could be detected by immunohistochemistry.

Our patient underwent pancreas resection because of the presence of a neuroendocrine tumor. On macroscopic examination, the tumor was 1.8 cm in maximum diameter (TNM staging, pT1). There was no vascular invasion present. None of the three lymph nodes was metastatic (TNM staging, pN0). The active proliferative rate of Ki-67 (a pathological grading marker) was 5%.

Well-differentiated neuroendocrine tumors of the pancreas usually show the characteristic nested, trabecular, or gyriform pattern known from neuroendocrine neoplasms of other organs. There are, however, architectural and cytological variations in some tumors, including abortive gland formation or oncocytic and clear cell changes.

In our case, the differential diagnosis of the biopsy specimen was focused on a distinction from other epithelial pancreas neoplasms especially the solid variants of acinar cell carcinoma, solid pseudopapillary neoplasm, as well as pancreatoblastoma. The latter tumor types may also exhibit the nesting pattern observed in our case. Therefore, the intensive homogenous staining pattern for synaptophysin in our case provided an important argument for the diagnosis of a neuroendocrine tumor. This diagnosis was further corroborated in the resection specimen by the lack of features defining acinar cell carcinoma (for example, acinar differentiation) or pancreatoblastoma or solid papillary neoplasm (for example, pseudopapillary growth pattern).

Our patient underwent a ^68^gallium-DOTATOC positron emission tomography (PET)/CT scan 4 months later, which did not show increased metabolic uptake. Seven months after pancreas resection his HbA1c increased from 6.9 to 8.7%. This led us to modify his diabetes medication to a combination including insulin glargine, insulin glulisine, and metformin.

## Discussion

This section discusses ADP and presents a systematic review of cases of pancreatic tumors in patients with ADP. Dorsal agenesis of the pancreas is a very rare congenital pancreatic malformation. Pancreatic development is a complex process and results from the fusion of the ventral and dorsal bud. The ventral bud forms the head and the uncinate process while the dorsal bud forms the upper part of the head, isthmus, and body tail of the pancreas [1]. Dorsal agenesis occurs when there is abnormal development of the dorsal pancreatic bud, but there is regular development of the ventral bud.

ADP is mostly asymptomatic, but common presenting symptoms include diabetes mellitus, abdominal pain, pancreatitis, enlarged pancreatic head, and, in a few cases, polysplenia [2].

The first description of this condition was published in 1911 in an autopsy finding [3]. Since then, there have been less than 100 reported cases in the literature. Our research in PubMed revealed 16 cases of pancreatic tumors among the very few reported cases of ADP (Table 1). This fact suggests a strong association between this rare congenital condition and the occurrence of pancreatic tumors. But we have to take in account that the number of cases of ADP may be higher, because not every case of ADP may be published. In addition, there is no systematic screening for this abnormality. Familial accumulation has been described which suggests a genetic predisposition. The exact genetic factor in humans has still not been established. However, a mutation in retinaldehyde dehydrogenase 2 (*Raldh2*) and gene *H1xb9* or deficiency of retinoic acid in mice studies leads to ADP [4, 5].

**Table 1:** Published cases of agenesis of the dorsal pancreas with associated pancreatic tumors

Reference	Age and gender	Symptoms	Histology	Imaging modality	Procedure
Saikaly *et al*., 2017 [9]	29, M	Abdominal pain	Infiltrating, moderately differentiated mucinous adenocarcinoma and cystic teratoma	MRCP	Whipple procedure (pyloric preserving) with total pancreatectomy, lymph node dissection, and chemotherapy
Cienfuegos *et al*., 2017 [10]	40, M	20-year history of chronic idiopathic cholestasis, hypercholesterolemia, type 2 diabetes mellitus	Atrophy of the exocrine pancreas and cystic cavities formed by transitional epithelium without cellular atypia	CT, MRCP	Total laparoscopic pylorus-preserving pancreatoduodenectomy
Nassif *et al.*, 2016 [11]	48, F	Incidental finding, asymptomatic	Well-differentiated, nonfunctioning neuroendocrine tumor	CT and MRI	Mass resection via spleen preserving laparoscopic approach
Mistry *et al*., 2015 [12]	42, M	Painless jaundice, type 2 diabetes mellitus	Ampullary carcinoma	CT	Pancreatoduodenectomy
Sannappa *et al*., 2014 [13]	51, F	Painless obstructive jaundice	Periampullary pancreaticobiliary adenocarcinoma	MRI, MRCP	Pancreatoduodenectomy
Oki *et al*., 2013 [14]	65, M	Back pain	Invasive ductal carcinoma	CT	Chemotherapy with 3 courses of gemcitabine and S1 followed by pancreaticoduodenectomy
Dumitraşcu *et al.*, 2012 [15]	44, F	Jaundice, epigastric pain	Well-differentiated tubular ductal adenocarcinoma	CT	Curative intent surgery pancreatoduodenectomy and adjuvant chemotherapy * in this case partial dorsal pancreas agenesis
Rittenhouse *et al*., 2011 [16]	37, F	Epigastric abdominal pain, known insulin-dependent diabetes mellitus	Moderately differentiated ductal adenocarcinoma	CT, EUS, ERCP	Pylorus-preserving resection of the pancreatic head and uncinate process, adjuvant chemotherapy with gemcitabine
Rittenhouse *et al*., 2011 [16]	59, F	Abdominal pain, weight loss, known insulin-dependent diabetes mellitus	Moderately differentiated ductal adenocarcinoma	CT	Pylorus-preserving resection of the pancreatic head and uncinate process, adjuvant gemcitabine-based chemotherapy and radiation
Rittenhouse *et al*., 2011 [16]	68, M	Elevated liver enzymes	Moderately differentiated ductal adenocarcinoma	None (intraoperative diagnosis)	Pylorus-preserving resection of the pancreatic head and uncinate process, adjuvant gemcitabine-based chemotherapy and radiation
Kapoor and Singh, 2011 [17]	55, M	Painless jaundice, pruritus, weight loss, cholangitis	Ampullary carcinoma	CT, intraoperative pancreatogram	Pancreaticoduodenectomy
Sakpal *et al*., 2009 [6]	49, M	Weight loss, fatigue, diarrhea	IPMN with well-differentiated, invasive mucinous adenocarcinoma	CT	Whipple procedure with total pancreatectomy, lymph node dissection
Ulusan *et al*., 2006 [18]	72, M	Jaundice, abdominal pain, hyperglycemia	Ductal adenocarcinoma	CT	Hepaticojejunostomy, cholecystectomy, and chemotherapy
Ulusan *et al*., 2005 [19]	49, F	Abdominal pain, hyperglycemia	Solid pseudopapillary tumor	Unknown	Whipple procedure
Nakamura *et al*., 2001 [20]	28, F	Asymptomatic	Solid papillary tumor	ERCP	Partial pancreatic head resection
Matsusue *et al*., 1984 [21]	53, F	Weight loss, abdominal pain	Ductal adenocarcinoma	CT	Total pancreatectomy, lymph node dissection

*CT* computed tomography, *ERCP* endoscopic retrograde cholangiopancreatography, *EUS* endoscopic ultrasound, *F* female, *IPMN* intraductal papillary mucinous neoplasm, *M* male, *MRCP* magnetic resonance cholangiopancreatography, *MRI* magnetic resonance imaging

Patients with ADP are mostly asymptomatic and ADP is detected during an evaluation for an unrelated cause, so that most cases of ADP are incidental findings. The clinical manifestation of ADP varies extremely from abdominal pain, pancreatitis, and diabetes mellitus to exocrine insufficiency with steatorrhea [6].

The association of pancreatic neoplasia and ADP has not been studied extensively, so the mechanism is unclear. Some theorized that ADP increases the risk of chronic pancreatitis, which in and of itself is a risk factor for pancreatic tumors. Our patient did not report any symptoms which can be associated with a chronic pancreatitis. An endoscopic ultrasound as well as CT scan did not show any sign of chronic pancreatitis. Gastroscopy before endoscopic ultrasound showed some ulcers, so that the hypothesis of a gastrin-producing tumor was favored. This hypothesis could not be confirmed by the immunohistochemistry of the tumor.

While the diagnosis of ADP was made at autopsy or laparotomy in the past, with the increasing availability of newer imaging techniques the diagnosis of ADP increased in the last decade. Diagnosis of ADP can be made by ultrasound, CT, magnetic resonance cholangiopancreatography (MRCP), and endoscopic retrograde cholangiopancreatography (ERCP). In the differential diagnosis, periportal lymphadenopathy should be considered as well as anatomic variations [7, 8].

We present a systematic review of pancreatic tumors associated with dorsal agenesis of the pancreas in Table 1. Twelve of the cases were malignant neoplasias, mostly ductal adenocarcinoma, one of them was a neuroendocrine carcinoma, and three of them a precancer. The median age at time of diagnosis was 49 years (range 28–72 years). Eight of the 16 published cases were men and eight were women. There is no trend of specific symptoms that leads to a diagnosis of ADP. Only six patients had an endocrine insufficiency that was associated with diabetes mellitus or hyperglycemia. Our patient reported that he had had diabetes mellitus type 2 for over 20 years.

## Conclusions

Here we present the 17th case of an ADP associated with a tumor. It is the second case report of an ADP-related neuroendocrine tumor. Our patient presented nonspecific abdominal symptoms: meteorism (bloating) and a subjective feeling of abdominal enlargement. A diagnosis of ADP was made with imaging techniques (CT and ultrasound). A diagnosis of neuroendocrine tumor in this setting needs histopathological or cytological confirmation, which can be done by a CT-guided biopsy or fine-needle aspiration via endoscopic ultrasound. If the tumor has been confirmed, then surgery is needed to resect the tumor.

The hypothesis about the association between pancreatic neoplasia and ADP leads us to the suggestion that every patient with diagnosed ADP should be observed with a focus on the early detection of potential malignancy.
